# Electrospun Bio-Scaffolds for Mesenchymal Stem Cell-Mediated Neural Differentiation: Systematic Review of Advances and Future Directions

**DOI:** 10.3390/ijms26199528

**Published:** 2025-09-29

**Authors:** Luigi Ruccolo, Aleksandra Evangelista, Marco Benazzo, Bice Conti, Silvia Pisani

**Affiliations:** 1 Department of Otorhinolaryngology, Fondazione IRCCS Policlinico San Matteo, 27100 Pavia, Italy; l.ruccolo@smatteo.pv.it (L.R.); a.evangelista@smatteo.pv.it (A.E.); marco.benazzo@unipv.it (M.B.); bice.conti@unipv.it (B.C.); 2 Integrated Unit of Experimental Surgery, Advanced Microsurgery, and Regenerative Medicine, Department of Clinical-Surgical, Diagnostic, and Pediatric Sciences, University of Pavia, Via Ferrata 9, 27100 Pavia, Italy; 3 Department of Drug Sciences, University of Pavia, Via Taramelli 12, 27100 Pavia, Italy

**Keywords:** neural tissue engineering, electrospun nanofibers, mesenchymal stem cells (MSCs), neural differentiation, biomaterial scaffolds

## Abstract

Neural tissue injuries, including spinal cord damage and neurodegenerative diseases, pose a major clinical challenge due to the central nervous system’s limited regenerative capacity. Current treatments focus on stabilization and symptom management rather than functional restoration. Tissue engineering offers new therapeutic perspectives, particularly through the combination of electrospun nanofibrous scaffolds and mesenchymal stem cells (MSCs). Electrospun fibers mimic the neural extracellular matrix, providing topographical and mechanical cues that enhance MSC adhesion, viability, and neural differentiation. MSCs are multipotent stem cells with robust paracrine and immunomodulatory activity, capable of supporting regeneration and, under proper stimuli, acquiring neural-like phenotypes. This systematic review, following the PRISMA 2020 method, analyzes 77 selected articles from the last ten years to assess the potential of electrospun biopolymer scaffolds for MSC-mediated neural repair. We critically examine the scaffold’s composition (synthetic and natural polymers), fiber architecture (alignment and diameter), structural and mechanical properties (porosity and stiffness), and biofunctionalization strategies. The influence of MSC tissue sources (bone marrow, adipose, and dental pulp) on neural differentiation outcomes is also discussed. The results of a literature search show both in vitro and in vivo enhanced neural marker expression, neurite extension, and functional recovery when MSCs are seeded onto optimized electrospun scaffolds. Therefore, integrating stem cell therapy with advanced biomaterials offers a promising route to bridge the gap between neural injury and functional regeneration.

## 1. Introduction

**Figure 1:**
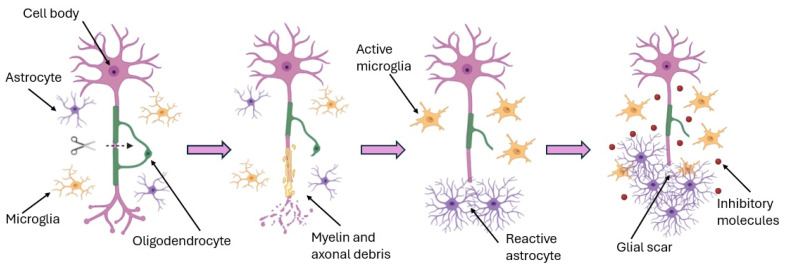
Schematic depiction of the central nervous system (CNS) response to injury. Astrocyte and microglia activation leads to the formation of a dense glial scar and the release of molecules that inhibit axonal regeneration, including chondroitin sulfate proteoglycans (CSPGs) and Nogo-A. Scientific illustrations were created using BioRender.com.

Injuries to the central nervous system (CNS), such as spinal cord injury, traumatic brain injury, and stroke, are typically devastating and result in lifelong neurological deficits. The adult CNS has very limited ability to regenerate damaged neurons or axons, due in part to the inhibitory environment that develops after injury (for example, glial scar formation and myelin-associated inhibitors) and the intrinsic lack of regenerative capacity in mature CNS neurons [1]. Figure 1 illustrates the complex biological response of the CNS to injury, including glial scarring and neuroinhibitory signaling, which collectively impair regeneration.

As a result, even partial spinal cord injuries can lead to the permanent loss of motor and sensory function, imposing heavy personal and societal burdens [2]. Traditional treatments are largely supportive and do not restore lost neural tissue, focusing instead on symptom management and rehabilitation. The absence of intrinsic regenerative capacity in the central nervous system, combined with inhibitory factors such as glial scar formation and pro-inflammatory environments, further complicates recovery. Consequently, there is a critical need for regenerative approaches capable of reconstructing the damaged neural architecture and re-establishing functional connectivity. Therefore, regenerative medicine strategies are being actively explored to bridge lesions, replace lost cells, and promote neural network reconnection [3].

Mesenchymal stem cells (MSCs) have gained attention as a potential therapeutic cell source for neural repair. MSCs are multipotent adult stromal cells obtainable from bone marrow, adipose tissue, Wharton’s Jelly, dental pulp, and other tissues. They can self-renew and differentiate into various cell types, and, importantly, they secrete a broad array of bioactive molecules that modulate the injury environment [4]. The ease of harvesting (often autologously) and their immunomodulatory, pro-regenerative secretory profile make MSCs especially attractive for CNS injury applications. Although MSCs are not neural stem cells, numerous studies have indicated that MSCs can be induced to display neuron-like or glial-like phenotypes under specific conditions (e.g., biochemical induction or co-culturing with neural cells) and can support endogenous neural regeneration through paracrine effects [5]. However, delivering MSCs alone into an injured neural tissue has limitations: the cells often have poor survival, may not remain at the injury site, and the hostile environment (inflammation, fibrosis) can impair their regenerative functions. For these reasons, scaffold-based approaches have been proposed as a means by which to overcome these issues by providing transplanted MSCs with a supportive, biomimetic microenvironment [6]. Electrospun biopolymer scaffolds are highly suitable for neural tissue engineering because of their nanometer-scale fibrous matrices that closely resemble the native extracellular matrix (ECM) of neural tissues. Electrospinning is a fabrication technique that uses an electrical field to draw polymer solutions (or melted polymer) into continuous ultra-thin fibers, which are collected into nonwoven meshes. The resulting fibrous scaffolds have a high surface-area-to-volume ratio and porosity, and their fiber diameters (often in the sub-micron to nanometer range) approximate the dimensions of collagen fibrils and other ECM fibers in neural tissue. This structural similarity can promote cell attachment and migration and provide contact guidance for extending neurites [7]. Additionally, the electrospinning process allows considerable control over scaffold architecture—including fiber alignment, diameter, mesh thickness, and mechanical properties—by adjusting parameters like the solution properties and spinning conditions. As a result, electrospun scaffolds can be tailored to meet the specific needs of neural tissue by making them sufficiently soft to match the spinal cord texture (≈1 kPa) and aligning fibers to guide axonal growth [8].

Central nervous system injuries have limited treatment options due to the poor regenerative capacity of neural tissue. Mesenchymal stem cells (MSCs) offer therapeutic potential through their paracrine and immunomodulatory effects, but their efficacy is limited when delivered alone. Electrospun scaffolds can overcome these limitations by mimicking the neural extracellular matrix, enhancing MSC survival, retention, and neural differentiation. This systematic review aims to critically assess recent advances in combining electrospun biopolymer scaffolds with MSCs for neural repair, identifying key design parameters and outcomes to guide future research.

This review aims to explore how electrospun biopolymer scaffolds can enhance the neuroregenerative potential of mesenchymal stem cells (MSCs) in central nervous system repair. We focus on how key scaffold features—such as fiber alignment, diameter, porosity, stiffness, and surface biofunctionalization—influence MSC behavior, including adhesion, survival, and neural differentiation. The review also compares MSCs from different tissue sources, examining their suitability for neural applications. Finally, we assess both in vitro and in vivo outcomes, highlighting trends, challenges, and future directions to guide the development of next-generation regenerative strategies.

## 2. Methods

### Literature Search Strategy

**Figure 2:**
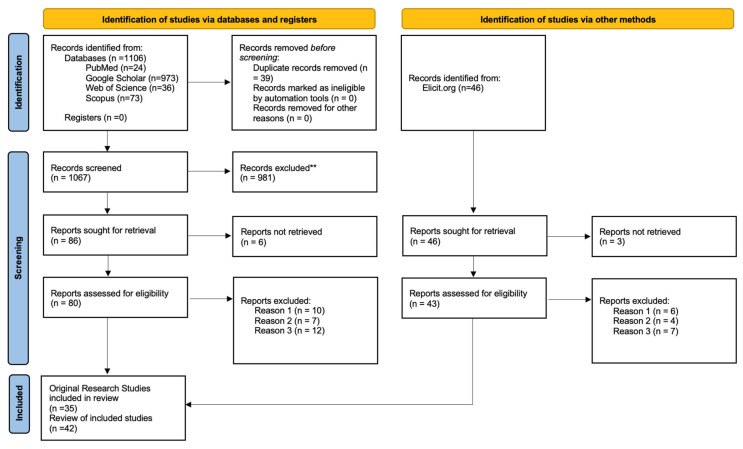
Systematic selection of studies following the PRISMA 2020 flow diagram for new systematic reviews which included searches of databases, registers, and other sources. The systematic search was conducted using five electronic databases: PubMed, Google Scholar, Web of Science, Scopus, and Elicit.org. A total of 1152 records were identified. ** Exclusion criteria included the following (**): Reason 1: Non-MSC cell sources (e.g., embryonic stem cells, iPSCs). Reason 2: Non-electrospun scaffolds (e.g., porous foams, freeze-dried matrices, and 3D-printed). Reason 3: Studies not involving the nervous system or neural outcomes.

A comprehensive literature search was conducted across five major academic sources: PubMed, Google Scholar, Web of Science (WoS), Scopus, and Elicit.org. The goal was to identify peer-reviewed articles focused on the application of electrospun biopolymer scaffolds in combination with MSCs for CNS tissue regeneration. The systematic selection of studies following the PRISMA 2020 flow diagram is reported in Figure 2.

Search terms included combinations of the following keywords:

“nanofibers”, “biomaterial”, “mesenchymal stem cells”, “neural”, and “central nervous system”. Boolean operators (AND, OR) were applied to refine queries depending on the platform.

To facilitate data management and screening, Publish or Perish 8 (PoP 8—8.18.5091.9307) software was used to batch export bibliographic metadata from Google Scholar, Scopus, PubMed, and WoS into a structured Excel spreadsheet. The Elicit.org semantic engine was queried separately using natural language questions (e.g., “Do mesenchymal stem cells and nanofibers support CNS regeneration?”) to identify conceptually related papers.

The initial search yielded 1152 records, of which 973 were from Google Scholar. After removing 39 duplicates, a total of 1113 unique entries were screened via their title and abstract. From these, 77 articles were selected for full-text review based on the following inclusion criteria:

- Original experimental research or reviews published.
- Use of MSCs derived from bone marrow, adipose tissue, dental pulp, or Wharton’s Jelly.
- Application of electrospun or nanofibrous scaffolds aimed at neural differentiation or CNS regeneration.
- In vitro or in vivo validation of scaffold–MSC interactions.
- Exclusion criteria included the following (**):
- Reason 1: Non-MSC cell sources (e.g., embryonic stem cells, iPSCs).
- Reason 2: Non-electrospun scaffolds (e.g., porous foams, freeze-dried matrices, and 3D-printed).
- Reason 3: Studies not involving the nervous system or neural outcomes.

Articles were further classified into experimental original research (35) or reviews (42), and organized according to scaffold material, MSC origin, fiber alignment, dimensional features, and the use of bioactive molecules or electrical stimulation. To assess the methodological quality and risk of bias in the included studies, a simplified evaluation strategy was applied, tailored to the heterogeneous nature of the literature (experimental and review articles). Each original research study (n = 35) was independently assessed by two reviewers by using a customized checklist based on key domains, including the completeness of outcome data, the consistency of experimental conditions, and reporting transparency (e.g., scaffold characterization, MSC source, and differentiation assays). Review articles (n = 42) were not subjected to formal bias scoring but were evaluated qualitatively based on the transparency of the methodology, the clarity of scope, and consistency with primary literature. This dual approach ensured a balanced and rigorous appraisal of the included evidence.

## 3. Results

### 3.1. Electrospun Bio-Scaffolds for Neural Regeneration: Composition and Properties

The electrospinning process enables the fabrication of fibrous scaffolds that mimic the neural ECM structure [9,10]. Aligned fibers are especially advantageous as they guide neurite extension and cell orientation, replicating developmental or peripheral nerve cues [11]. Although random fibers conform better to irregularly shaped lesions, they provide no directional guidance [12]. Fiber diameter also plays an important role: nanoscale fibers enhance cell attachment and differentiation by resembling a native ECM, but very small diameters may reduce pore size, hindering cell infiltration [13]. To address this, combining nano- and microscale fibers creates hierarchical scaffolds that support both surface interaction and 3D cell migration [14]. Synthetic polymers such as polycaprolactone (PCL), poly(lactic acid) (PLA), poly(lactic-co-glycolic acid) (PLGA), thermoplastic polyurethane (TPU), and polyvinylidene fluoride (PVDF) are widely employed in neural tissue engineering, particularly in nanofiber form, due to their favorable mechanical strength, controlled degradation rates, biocompatibility, and manufacturing reproducibility. These materials can effectively guide mesenchymal stem cells (MSCs) toward neuronal lineages. However, their bioinert nature necessitates surface functionalization or blending with bioactive components to improve biointegration and support neuroinductive signaling [15].

Poly-ε-caprolactone (PCL) is one of the most frequently employed synthetic polymers in neural tissue engineering due to its processability via electrospinning and slow in vivo degradation, which can extend by up to 24 months [7,8,11,16,17,18,19,20,21,22,23,24,25,26,27,28,29,30,31,32,33,34,35,36,37]. Aligned nanofibrous PCL mats exhibit a Young’s modulus of approximately 10 MPa [38], offering structural robustness over time. Despite these advantages, PCL is highly hydrophobic (contact angle > 90°), which hampers initial cell adhesion and spreading. To address this limitation, various surface modification strategies have been explored. For instance, plasma treatment has been shown to significantly reduce the water contact angle and improve mesenchymal stem cell (MSC) adhesion [30].

Poly(lactic-co-glycolic acid) (PLGA) is characterized by its controllable degradation profile, which can be tuned by adjusting the ratio of lactide to glycolide. For instance, in Mohammadalizadeh et al. [39], PLGA with a 75:25 ratio enabled a sustained release of brain-derived neurotrophic factor (BDNF;) however, its acidic hydrolysis led to a significant local pH drop. This side effect was mitigated by incorporating a buffering hydrogel (alginate), which maintained physiological conditions for over four weeks. PLGA is thus well-suited for applications requiring the timed delivery of neurotrophic factors, although its in vivo use demands careful consideration to prevent acidic toxicity and secondary inflammation.

Poly(lactic acid) (PLA)/Poly(L-lactic acid) (PLLA), while sharing many characteristics with PLGA, exhibits greater stiffness, a higher tendency to crystallize, and higher hydrophobicity, making it more suitable for scaffolds that require structural integrity or precise spatial orientation. Aligned PLLA fibers have been shown to effectively guide mesenchymal stem cell (MSC) alignment and promote neurite outgrowth. However, its inherently low electrical conductivity poses a limitation, which has been partially addressed by incorporating carbon nanotubes (CNTs) or graphene oxide (GO) [40,41].

Polyvinylidene fluoride (PVDF) is a non-degradable and biocompatible piezoelectric polymer capable of generating microcurrents in response to mechanical or magnetic stimuli. Li et al. [42] developed PVDF-based scaffolds incorporating cobalt ferrite (CoFe_2_O_4_) nanoparticles, which, when exposed to an alternating magnetic field, led to a 73% increase in Tuj1 expression and a reduction in glial inflammation. In the same study, PVDF scaffolds embedded with ferric oxyhydroxide (FeOOH) and activated by ultrasound stimulation released Fe^3+^ ions, promoting the formation of organized neural networks even in the absence of exogenous growth factors.

Thermoplastic polyurethane (TPU) has recently emerged as a promising material due to its elasticity and compatibility with the electrospinning process. Pouladzadeha et al. [43] developed TPU-based scaffolds enriched with CNTs, which promoted neuronal alignment and neurite outgrowth even in the absence of electrical stimulation, highlighting the synergistic role of topographical and conductive cues.

These studies demonstrate that several synthetic polymers, when appropriately combined with bioactive and conductive elements, can mimic the neural microenvironment and guide MSCs towards a desired neuronal lineage.

Natural polymers such as collagen, gelatin, chitosan, silk fibroin, and hydrophilic polysaccharides offer native biocompatibility and biochemical signals that support stem cell adhesion, survival, and differentiation, features often lacking in synthetic scaffolds [44]. However, their limited mechanical robustness, processability, and aqueous stability necessitate careful modification or hybridization for neural tissue applications.

Collagen, the predominant ECM protein in the CNS, is frequently employed to replicate neural microarchitecture. Bagher et al. [16] showed that type I collagen scaffolds support the adhesion and spreading of Wharton’s Jelly-derived MSCs (WJ-MSCs). Yet, their low viscosity and rapid hydrolytic degradation hinder electrospinning. They are often crosslinked or coated onto sturdier synthetic fibers to compensate for their structural limitations.

Gelatin, a collagen derivative, is more soluble and electrospinnable, while preserving adhesive motifs like Arginine-Glycine-Aspartic acid (RGD). According to Biazar et al. [45], functionalization with laminin-derived peptides (e.g., IKVAV) enhanced MSC migration and neuronal differentiation, making it a versatile base for biofunctional scaffolds.

Chitosan, a positively charged biopolymer derivative from chitin, interacts electrostatically with membranes and trophic factors. Boroojeni et al. [46] developed aligned PCL/PLGA/chitosan scaffolds that enhanced neurogenesis through synergistic topographical and biochemical cues. Rahimzadegan et al. [22] further improved scaffold performance by enriching chitosan matrices with gold nanoparticles and hyaluronic acid, boosting βIII-tubulin expression, porosity, and wettability.

Silk fibroin, though less commonly used, has a β-sheet structure that offers excellent mechanical performance and slow degradation, as shown by Raspa et al. [47]. When combined with conductive agents like graphene oxide, it acquires electroactive properties, supporting strategies that couple material design with neuromodulation [25].

Alginate and hyaluronic acid, although rarely electrospun alone, are widely used as hydrogel matrices or coatings. Tang et al. [15] described their role in improving scaffold hydration, buffering acidic byproducts, and maintaining stem cell viability. Within multiphase composites, these polysaccharides act as biochemical modulators in tandem with synthetic structural elements.

In summary, natural polymers uniquely influence neural stem cell fate via endogenous signaling mechanisms. Their clinical translation, however, requires integration with robust carriers or advanced functionalization strategies to overcome inherent mechanical and stability constraints.

Fiber alignment is a key topographical cue influencing MSC behavior on electrospun scaffolds. Aligned nanofibers mimic the linear architecture of axonal tracts in the CNS, whereas randomly oriented fibers lack such directional guidance. Even in the absence of biochemical additives, aligned fibers’ topography has been shown to induce MSC polarization and the extension of neurite-like processes along the fiber axis. Upon contact with aligned fibers, MSC cytoskeletal filaments reorganize longitudinally, leading to elongated morphologies and the activation of mechanotransduction pathways that favor neuronal lineage commitment [11]. Several studies demonstrate that MSCs cultured on aligned fiber mats significantly upregulate neuronal-specific markers, such as βIII-tubulin (Tuj1), MAP2, and NEUN, compared to those on random fibers. For instance, human umbilical cord-derived MSCs (hUC-MSC) grown on aligned polypyrrole/polylactide (PPy/PLA) nanofibers under electrical stimulation exhibited increased expression of NF-L and Nestin, and developed organized neurite bundles aligned with the scaffold structure [13]. Advanced fabrication techniques allow precise control over fiber orientation. Rotating drum collectors, parallel electrodes, and magnetic field-assisted electrospinning can generate highly aligned or patterned fiber regions. Notably, magnetic field-assisted wet electrospinning has enabled the creation of 3D scaffolds with distinct aligned and random regions within the same construct, supporting both MSC infiltration and organized neural network formation. Fibers’ diameter and pore size are equally critical. Nanofiber diameters ranging from 300 to 500 nm closely resemble native axons and extracellular matrix fibrils and have been associated with enhanced neuronal marker expression in MSCs. However, excessively small diameters can reduce scaffold porosity, hindering cell migration and neurite ingrowth. In contrast, microscale fibers (≥1 µm) promote better cellular infiltration and 3D network development but may be less efficient at inducing early neurogenic markers [48]. Techniques such as mixed-diameter electrospinning can balance nanoscale features with sufficient porosity for effective cell migration. In summary, the physical structure of electrospun nanofibers, including their alignment, diameter, and spacing, plays an active role in directing MSC fate. Engineering fiber topography to replicate the aligned, porous architecture of neural tissue is a powerful strategy with which to induce neuronal differentiation via mechanobiological signaling.

Beyond passive structural cues, scaffolds can be designed to deliver electrical or mechanical stimuli to cells [49]. The nervous system is inherently electroactive [50]; thus, incorporating conductive elements into scaffolds can better replicate its microenvironment. Conductive polymers (CPs), such as polyaniline (PANI), polypyrrole (PPy), and Poly-3,4-ethylenedioxythiophen (PEDOT), as well as carbon-based nanomaterials like graphene and CNTs, have been successfully electrospun into fibrous matrices to provide such functionality [43,51,52,53]. These scaffolds enable the application of external electrical stimulation (ES), which has been shown to significantly enhance neural differentiation. For example, the daily application of a mild electric field (115 V/m, 100 Hz, 1 min/day for 3 days) to conjunctiva-derived MSCs cultured on PCL/PPy nanofibers induced the robust upregulation of Nestin (~127-fold), β-tubulin (~30-fold), and MAP2 (~52-fold), indicating successful neurogenic differentiation [36]. Three-dimensional conductive architectures offer added benefits. A dual-nozzle electrospinning strategy was used to fabricate PANI-blended fibers into an oriented 3D scaffold, enhancing both neurite outgrowth and the maturation of neural morphology in stem cells compared to non-conductive controls [54]. Magnetoelectric scaffolds composed of PVDF combined with CoFe_2_O_4_ nanoparticles can generate electrical signals under external magnetic fields. This magnetostrictive/piezoelectric coupling mimics neuronal signaling and supports the neural differentiation of MSCs without wired stimulation. In vitro, these hybrid membranes significantly enhanced Tuj1 expression and reduced the astrocytic marker GFAP, indicating a shift toward neuronal fate. Additionally, piezoelectric scaffolds made from PVDF integrated with FeO(OH) nanoparticles generate transient electric signals when stimulated by low-intensity ultrasound. This mechano-electrical stimulation led to Ca^2+^ influx and PKC-MAPK pathway activation in human MSCs, promoting neural differentiation even in the absence of added growth factors [3]. These findings underscore how electroactive and piezoelectric scaffolds act as more than structural matrices. They dynamically participate in directing stem cell fate, supporting the concept of “electroceutical” implants capable of modulating regeneration via non-invasive external fields.

**Figure 3:**
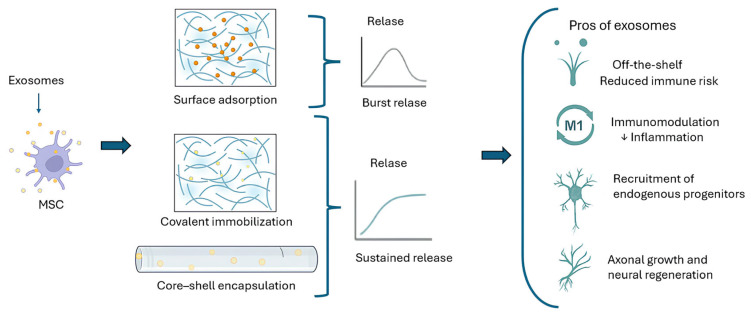
MSC-derived exosomes can be integrated into electrospun scaffolds by surface adsorption, covalent immobilization, or core–shell encapsulation, leading to distinct release profiles. Once released, exosomes provide immunomodulatory, neuroprotective, and regenerative effects, offering a promising acellular alternative to live MSC transplantation. ↓ indicate the exosomes.

While topography and conductivity address physical cues, the biochemical functionalization of scaffolds provides instructive molecular signals to stem cells. A common strategy is to decorate fiber surfaces with motifs derived from ECM proteins or other bioactive ligands. Laminin-derived peptides, such as the IKVAV (Isoleucine–Lysine–Valine–Alanine–Valine) sequence from laminin or RGD peptides (found in fibronectin), can be covalently bonded or adsorbed to nanofibers. These peptides engage cell adhesion receptors (integrins) on MSCs, activating intracellular pathways that promote neural differentiation. For example, PCL nanofibers functionalized with IKVAV peptides significantly increased neuronal morphology and βIII-tubulin/MAP2 expression in MSCs, with even greater effects when the fibers were also aligned [55]. The cooperative effect of biochemical signals (IKVAV) with physical cues (alignment) yielded the highest induction of neuron-like cells. Other groups have achieved similar results using RGD or whole adhesive proteins like fibronectin and laminin coated on fibers, all aiming to simulate the native neural niche chemistry [54]. Neurotrophic factors can likewise be incorporated. Nerve growth factor (NGF), brain-derived neurotrophic factor (BDNF), glial cell line-derived neurotrophic factor (GDNF), and others have been immobilized on or within electrospun scaffolds to create sustained local release. MSCs on nanofibers loaded with BDNF/NGF show improved survival and stronger neural marker expression due to the continuous trophic support. Importantly, binding these growth factors to a scaffold can establish gradients akin to those in development. For example, an electrospun fiber gradient with a high NGF concentration on one end can guide neuron-like differentiation and neurite extension directionally, as MSCs migrate up the NGF gradient [56]. A novel biochemical approach is the use of MSC-derived exosomes within scaffolds. Exosomes are nano-sized vesicles secreted by cells, carrying proteins, mRNAs, and microRNAs that modulate cell behavior and replicate the many therapeutic effects of MSCs, such as neuroprotection and immunomodulation, without requiring cell transplantation. To date, different strategies have been developed to incorporate exosomes into electrospun fibers, including surface adsorption, covalent immobilization, and core–shell encapsulation. These loading methods critically affect release kinetics, ranging from rapid burst release to sustained, long-term delivery, which is essential for the stable modulation of the injury microenvironment. In preclinical models of traumatic brain injury, dual-exosome-loaded scaffolds (MSC- and NSC-derived) reduced neuroinflammation and enhanced neuronal marker expression (Tuj1, MAP2), while also recruiting endogenous progenitors [13]. Core–shell designs, in particular, protect exosomes during fabrication and allow the directional, prolonged release of bioactive factors, maintaining therapeutic levels for weeks [57]. A schematic summary of these approaches is presented in Figure 3.

Despite encouraging results, several challenges remain. Loading efficiency varies greatly among fabrication strategies, and while exosomes can reproduce many of the trophic and immunomodulatory effects of live MSCs, their efficacy in driving long-term neuronal differentiation and network integration is more limited. Nevertheless, exosomes offer distinct translational advantages, including reduced tumorigenic risk, lower immunogenicity, and simplified storage logistics, making them attractive as complementary or even alternative tools to cell transplantation, especially when combined with electrospun scaffolds that provide controlled spatial and temporal release. In summary, biochemical functionalization turns a passive scaffold into an instructive microenvironment. By presenting cell-adhesive ligands, soluble factor delivery, or even cell-derived vesicles, the scaffold actively signals MSCs to survive, migrate, and commit to neural phenotypes. Future scaffolds are likely to integrate multiple biochemical cues—for instance, aligned, IKVAV-coated fibers that slowly release BDNF and even specific microRNAs to maximize neuroregeneration. This multi-modal approach could dramatically amplify the neurogenic potential of MSCs, bridging the gap between cell therapy and tissue engineering.

**Figure 4:**
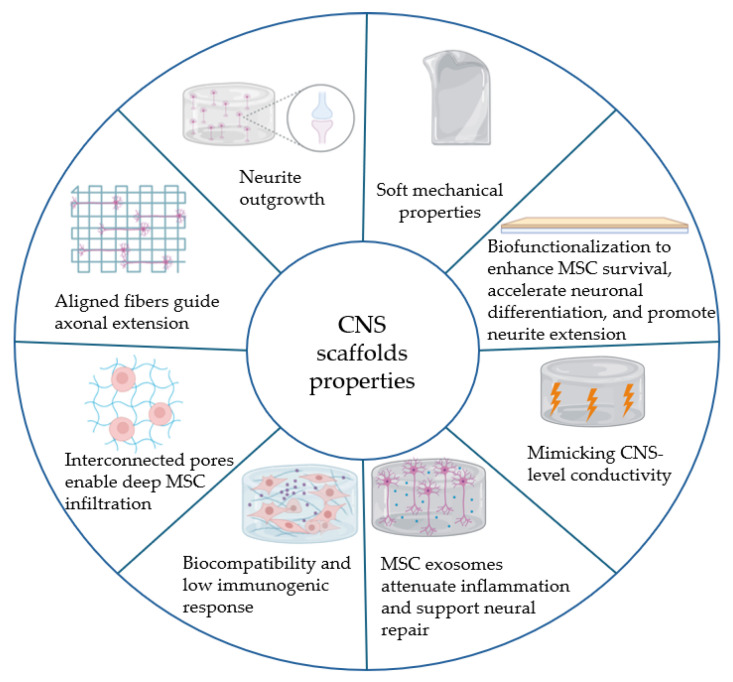
Schematic overview of the eight interdependent features of electrospun biopolymer scaffolds designed for MSC-mediated regeneration of the central nervous system. Scientific illustrations in this work were created using BioRender.com.

The mechanical characteristics of a scaffold are another critical design factor, particularly stiffness (elastic modulus). Neural tissue like the brain and spinal cord is extremely soft (storage modulus in the order of 0.1–1 kPa). If a scaffold is too stiff relative to the host tissue, it can cause mechanical mismatch, glial scarring, and even direct cells towards non-neural fates (e.g., osteogenesis). For MSC-based neural applications, studies indicate that substrates with a modulus in the soft range (<1–10 kPa) support neuronal differentiation, whereas very rigid substrates (>20–30 kPa) push MSCs towards osteogenic or glial lineages [58]. Electrospun fibers of commonly used synthetic polymers are often relatively stiff (PCL mats can be several MPa in modulus). To soften the microenvironment, researchers have developed hybrid scaffolds that combine electrospun fibers with hydrogel matrices. In these composites, the fibrous scaffold provides tensile strength and guidance, while the interpenetrating hydrogel (e.g., collagen, alginate, and self-assembling peptide hydrogel) provides tissue-like softness and high water content [38]. One example is an electrospun PCL/chitosan mesh embedded in an alginate hydrogel. This hybrid scaffold retained the aligned fibrous structure (for guidance) but had a bulk stiffness an order of magnitude lower than the pure fiber mat. In vitro, MSCs on the soft hybrid showed greater neurite extension and expressed higher βIII-tubulin and MAP2 than on the stiff fiber mat alone [59]. The alginate hydrogel not only softened the matrix but also provided bioactive cues and improved nutrient diffusion, resulting in healthier, more neuronal-like MSCs. Similarly, incorporating a physically crosslinked gelatin or collagen hydrogel with electrospun fibers can mitigate the acidic byproducts of polymer degradation (e.g., PLGA releases lactic acid) and reduce inflammatory responses. Hydrogels can also be loaded with anti-inflammatory drugs or factors like IL-10 to locally counteract any immune reaction to the scaffold [60]. Such smart designs improve implantation feasibility and reduce additional tissue damage during surgery. In conclusion, the mechanical tuning of electrospun scaffolds, whether by choosing softer polymers, blending with hydrogels, or using smart materials, is essential to create a permissive environment for neural regeneration. The most promising platforms tend to be composites that achieve an optimal balance: they possess the aligned, fibrous structure needed for axon guidance and MSC morphology cues, yet they are soft and hydrated enough to integrate with host tissue and avoid triggering foreign body responses. For instance, a recent composite scaffold of aligned fibers within a viscoelastic hydrogel was implanted in a spinal injury model and showed minimal glial scar formation alongside robust axonal ingrowth [59]. By integrating mechanical biocompatibility with the earlier mentioned biochemical and topographical features, modern electrospun scaffolds can be multifunctional platforms that address the multifaceted challenges of CNS regeneration. A summary of the ideal properties required for CNS scaffolds is presented in Figure 4. Soft mechanical properties mimic native CNS tissue elasticity, while biofunctionalization enhances MSC survival, accelerates neuronal differentiation, and drives neurite extension. Electrical conductivity tuned to the CNS level delivers optimal stimulation, and MSC-derived exosomes mediate local immunomodulation to attenuate inflammation and support repair. Inherent biocompatibility with low immunogenicity ensures seamless integration, and interconnected porosity together with aligned fiber architecture fosters deep cell infiltration, three-dimensional network formation, and guided axonal outgrowth.

### 3.2. Mesenchymal Stem Cells (MSCs) in Neural Regeneration

MSCs play a central role in neural regeneration through their ability to partially differentiate into neural cells, modulate immune responses, and most importantly secrete a rich array of regenerative factors. This paracrine activity is considered the main driver of their therapeutic effect on neural repair [61]. MSCs release neurotrophic factors such as BDNF, NGF, VEGF, IGF-1, and NT-3, which promote neuronal survival and neurite extension, along with anti-inflammatory cytokines like IL-10 and TGF-β1, which help counteract post-injury inflammation. Evidence suggests that the benefits of MSC therapy in spinal cord or brain injury are primarily due to these secreted factors rather than direct cell replacement. For instance, MSC-conditioned medium has been shown to reproduce many neuroprotective effects in vitro, including protection against glutamate toxicity and enhanced neurite outgrowth [62]. Embedding MSCs into scaffolds enables the localized, sustained delivery of these bioactive molecules, improving outcomes in animal models of spinal cord injury by reducing lesion size and preserving axons [63]. An advanced strategy involves using MSC-derived exosomes, small extracellular vesicles capable of crossing the blood–brain barrier and delivering microRNAs like miR-133b and miR-7. These miRNAs enhance neural plasticity and activate endogenous neural progenitors, further supporting repair [23]. Overall, by harnessing MSCs’ potent paracrine signaling, either through direct integration or exosome delivery, electrospun scaffold systems can create a regenerative microenvironment highly conducive to neural healing.

One of the most important properties of MSCs in neural repair is their immunomodulatory capacity, which is crucial given that inflammation and glial scarring significantly hinder CNS regeneration. MSCs exert these effects through multiple mechanisms: they secrete anti-inflammatory cytokines such as IL-10 and TGF-β, and promote the shift of macrophages/microglia from a pro-inflammatory (M1) to a pro-regenerative (M2) phenotype [64]. MSCs can also inhibit immune cell activity, suppressing T and B cell proliferation, as well as dampening NK cell and dendritic cell responses. This immune modulation leads to reduced tissue swelling, less secondary damage, and smaller glial scars due to decreased deposition of inhibitory molecules by reactive glia. In spinal cord injury (SCI) models, MSC transplantation lowered pro-inflammatory interleukins and increased arginase-1 expression (an M2 marker), correlating with reduced glial scarring. MSCs are also hypoimmunogenic, particularly when allogeneic and lacking MHC-II, allowing for low-rejection implantation. They respond to inflammatory signals (e.g., SDF-1) by migrating toward lesion sites, where they function as a biological “drugstore,” delivering anti-inflammatory and trophic factors [57]. This immunomodulatory role has been confirmed in both rodent models and early clinical trials, where MSCs were associated with reduced astrogliosis and better neural tissue preservation compared to controls. Scaffold integration enhances these effects, helping retain MSCs at the injury site and enabling the co-delivery of anti-inflammatory agents. For example, a peptide-functionalized scaffold that neutralized IL-6, when combined with MSCs, significantly reduced chronic inflammation in a traumatic brain injury model, resulting in improved neuronal survival [40].

MSCs have the ability to differentiate into neuron-like and glial-like cells under appropriate conditions. This neurodifferentiation is not as efficient or stable as that of neural stem cells, but it is well documented. Chemical cues such as retinoic acid, β-mercaptoethanol, or growth factors (BDNF, EGF) can induce MSCs to express neuronal markers (e.g., βIII-tubulin, NeuN) and even adopt neuron-like morphologies in vitro. For instance, human BM-MSCs exposed to a cocktail of neurotrophic factors and cyclic AMP for 7 days extended neurite-like processes and expressed MAP2 and NSE, indicating a partial neuronal phenotype [62]. However, without a supportive microenvironment (like a scaffold or co-culture), these induced neural phenotypes can be transient. This is where scaffolds play a crucial role: they provide physical and biochemical cues to stabilize and enhance differentiation. Simply culturing MSCs on an appropriate scaffold can itself spur neural differentiation even without exogenous chemicals. A telling study compared hMSCs from bone marrow and endometrium on electrospun PCL fibers: both cell types differentiated into motor neuron-like cells on the scaffold when given basic neural induction media, expressing markers such as HB9 and neurofilament-H [46]. The 3D fiber context supported neuronal survival and outgrowth, and both MSC sources showed comparable neurogenic capability on the fibers. It is important to note that MSC-to-neuron differentiation in vivo remains less efficient, and often only a small percentage of transplanted MSCs may exhibit neural markers. Therefore, the current consensus is that MSCs aid neural repair more through trophic and immunomodulatory effects, with differentiation playing a secondary role. Nonetheless, even a minor fraction of MSCs turning into neuronal or oligodendroglial cells could contribute to replacing lost cells, especially when combined with factors that improve their maturation (e.g., miR-7 delivery) [23]. MSCs can be isolated from various adult tissues, and their properties (proliferation rate, secretome, and differentiation bias) can differ depending on the source. Here we compare key MSC sources used in neural tissue engineering, focusing on bone marrow, adipose tissue, dental pulp, and others—to see how origin affects neural regeneration outcomes. Each subtype has its pros and cons in the context of scaffolds for CNS repair.

#### 3.2.1. Bone Marrow MSCs (BM-MSCs)

Bone marrow-derived mesenchymal stem cells (BM-MSCs) are among the most extensively investigated cell sources in neural regenerative medicine due to their ability to differentiate into neuronal phenotypes, their relative ease of isolation, and their established clinical safety profile. In a study by Ji et al. [54], supramolecular nanofibers composed of peptide amphiphiles bearing the laminin-mimetic IKVAV sequence successfully induced the neural transdifferentiation of BM-MSCs. After two weeks of culture, BM-MSCs exhibited the upregulation of neuronal markers such as TUJ-1, MAP2, and NEUN, along with a polarized cytoskeletal architecture and neuron-like morphology. Similarly, Pei et al. [65] demonstrated that BM-MSCs embedded in hydrogel/nanofiber composite scaffolds significantly ameliorated ischemic brain injury in vivo. The treatment reduced brain edema and infarct volume while improving neurological outcomes. Mechanistically, the BM-MSC-loaded scaffolds downregulated exosomal miR-206 levels, leading to the activation of the PI3K/AKT signaling pathway. In a 3D context, Shirian et al. [7] reported that BM-MSCs cultured on electrospun poly(ε-caprolactone) (PCL) scaffolds and exposed to neural inductive cues differentiated into motor neuron-like cells, expressing lineage-specific markers such as HB9, Islet-1, and neurofilament-H. Furthermore, Sun et al. [3] developed a multifunctional hydrogel composed of chitosan and self-assembling peptide nanofibers, which supported BM-MSC proliferation and migration, while modulating the inflammatory microenvironment following spinal cord injury. The hydrogel system effectively promoted endogenous neurogenesis and axonal regeneration in vivo. Collectively, these studies underscore the therapeutic potential of BM-MSCs in supporting neuroprotection, neuronal differentiation, and axonal repair across a range of central nervous system injury models.

#### 3.2.2. Adipose-Derived MSCs (AD-MSCs)

Adipose-derived mesenchymal stem cells (AD-MSCs) are an attractive cellular source for neural regeneration due to their abundance, minimally invasive harvest, and responsiveness to biochemical and topographical cues. Several experimental studies have shown that AD-MSCs can commit to neuronal-like phenotypes when cultured on functional scaffolds in the presence of appropriate differentiation stimuli. Pinar et al. [66] investigated the behavior of human AD-MSCs isolated from epidural and subcutaneous adipose tissue and cultured them on electrospun poly(ε-caprolactone)/graphene oxide (PCL/GO) scaffolds. To induce neuronal differentiation, the cells were treated with a specialized neurogenic medium containing BDNF (10 ng/mL), EGF (20 ng/mL), bFGF (20 ng/mL), IBMX (0.5 mM), and a neural stem cell proliferation supplement (2%). After 15 days, the AD-MSCs exhibited neurite-like outgrowths, elevated acetylcholinesterase activity, and significant upregulation of the neuronal markers βIII-tubulin and MAP2, indicating progression toward a functional neuronal phenotype. In a complementary approach, Borah et al. [67] cultured primary human AD-MSCs on conductive nanofibers composed of polyaniline and chitosan, which were surface-functionalized with tannic acid to enhance cell adhesion and biointerface performance. These cells were exposed to a simplified neuronal differentiation medium containing only bFGF and EGF. After 14 days, more than 85% of the cells were positive for βIII-tubulin, and approximately 40% expressed GFAP, supporting a mixed neuronal–glial commitment. Additionally, the increase in urease enzymatic activity under differentiation conditions suggested a metabolic component linked to the neuronal maturation process. In preclinical and clinical overviews, reviews such as those by Huang et al. [68] and Raspa et al. [25] summarize multiple experimental studies in which AD-MSCs were transplanted into models of spinal cord injury (SCI). These studies consistently report that the therapeutic effects of AD-MSCs are primarily mediated through paracrine mechanisms, rather than direct neuronal replacement. Specifically, AD-MSCs have been shown to secrete neurotrophic factors such as NGF, BDNF, and VEGF, modulate inflammation, support axonal preservation, and reduce cavitation in the injured spinal cord tissue. These properties contribute to the neuroprotective and immunomodulatory potential of AD-MSCs, highlighting their relevance as a supportive cell type for enhancing neural tissue repair in vivo.

#### 3.2.3. Dental Pulp Stem Cells (DPSCs)

Dental pulp stem cells (DPSCs), derived from the neural crest, exhibit a strong affinity for the neuroectodermal lineage, making them particularly promising for neural tissue regeneration strategies. Their intrinsic plasticity enables differentiation into neuron-like cells, astrocytes, and oligodendrocytes, especially when exposed to appropriate neuroinductive stimuli. Liu et al. [5] reported that treatment with neurotrophic factors such as NGF, bFGF, forskolin, and retinoic acid induces the expression of both early and mature neuronal markers in DPSCs, including Nestin, βIII-tubulin, MAP2, and GFAP. Moreover, DPSCs secrete significant levels of neurotrophic factors such as BDNF, GDNF, and NT-3, which play key roles in neuroprotection, axonal regeneration, and modulation of the inflammatory microenvironment in experimental spinal cord injury (SCI) models. In these models, DPSC transplantation led to improved motor recovery, increased GAP-43 expression (associated with axonal outgrowth), and reduced GFAP levels, indicating the attenuation of reactive astrogliosis. Schepici et al. [69] highlighted the importance of serum-free culture conditions and scaffold architecture in promoting the neuronal differentiation of DPSCs. Specifically, the use of aligned electrospun nanofibers combined with a medium enriched with EGF, bFGF, and retinoic acid significantly increased the expression of neuronal markers (Nestin, βIII-tubulin, and MAP2), along with polarized cell morphology and extended neuritic processes. This underscores the synergistic effect of chemical stimulation and topographical cues in directing neurogenesis. Nanotopographical features of the fibers also play a critical role. As shown by Xue et al. [8], DPSCs cultured on aligned nanofibers exhibited polarized morphology and the upregulation of early neuronal markers, suggesting that substrate topography alone can profoundly influence differentiation trajectories. Finally, Wang et al. [70] developed a coaxial core–shell electrospun scaffold, with NGF in the shell and BMP-2 in the core, capable of simultaneously inducing neurogenic and osteogenic differentiation in DPSCs. In the presence of this scaffold, the cells activated the mTOR and Erk1/2 signaling pathways and showed strong expression of MAP2 and βIII-tubulin. This approach demonstrates how spatial and molecular integration within multifunctional scaffolds can enhance the neuroregenerative potential of DPSCs. Overall, DPSCs represent a highly plastic and environmentally responsive mesenchymal population, whose neuronal differentiation capacity can be substantially enhanced through the use of engineered biomaterials and the targeted modulation of trophic factors.

#### 3.2.4. Other MSC Sources (Wharton’s Jelly, Conjunctiva, etc.)

Beyond the big three, researchers have explored MSCs from birth-associated tissues or unusual locations. Wharton’s Jelly MSCs (WJ-MSCs) from the umbilical cord are very potent proliferators and immunomodulators, and they are neonatal so have “young” characteristics. They have been used on nanofiber scaffolds with success; for example, one study grew WJ-MSCs on aligned PLLA nanofibers and found the cells aligned and expressed NF200 and GFAP, indicating a mix of neuronal and glial differentiation. WJ-MSCs also secrete abundant exosomes; one can envision using WJ-MSCs or their exosomes in a scaffold for perinatal brain injury treatments. Conjunctiva MSCs (from the eye conjunctival tissue) were recently described and applied to conductive scaffolds. These CJ-MSCs responded strongly to electrical stimulation, showing optimal neural differentiation at a specific stimulation regimen [71]. They might be of interest due to their developmental origin (the eye’s mesenchyme has some neural crest contribution). Olfactory mucosa MSCs (sometimes termed olfactory ecto-MSCs) are another niche, given their location in the nasal cavity; they are in a regenerative, neural-rich environment. Indeed, MSCs from olfactory tissue were used in the Bakhtiary et al. [61] 3D scaffold study and showed excellent neural differentiation and migration in that 3D scaffold.

To provide an up-to-date and comparative perspective on this rapidly evolving field, Table 1 summarizes 35 key experimental selected studies that investigated the neural differentiation of mesenchymal stem cells (MSCs) from various tissue sources using electrospun biomaterial scaffolds. The table highlights the type and properties of each scaffold, the origin of MSCs, differentiation protocols, outcomes on cell adhesion/proliferation, neural differentiation results, and the corresponding reference. This overview is intended to guide readers through the diversity of current approaches and facilitate direct comparison of experimental outcomes.

Among the 35 studies reviewed, only 43% reported quantitative mechanical characterization of the scaffolds (e.g., tensile strength, Young’s modulus, and strain at break). This is a significant gap, considering that mechanical properties are critical in neural tissue engineering to ensure structural compatibility with soft neural tissues (moduli in the 0.1–10 kPa range for the spinal cord or brain). From a materials perspective, no single biomaterial emerges as clearly preferred: a wide variety of synthetic (e.g., PCL, PLA, and PVDF), natural (e.g., gelatin, collagen), and composite or functionalized systems (e.g., with SPIONs, exosomes, and electrical conductivity) have been employed. This reflects the ongoing exploration of multifunctional scaffolds rather than convergence on a standard formulation. In terms of MSC source, the majority of studies used human-derived MSCs (from bone marrow, adipose tissue, Wharton’s Jelly, etc.), with fewer using rodent MSCs. This trend likely reflects a growing interest in translational relevance and clinical applicability.

### 3.3. In Vivo and Clinical Trial Evidence

In the face of the intrinsic complexity of CNS injuries and its limited capacity for self-repair, preclinical investigations in animal models represent a critical proving ground for the efficacy of novel therapeutic strategies based on electrospun and hybrid scaffolds [77]. These studies not only assess the regenerative potential of biomaterials in functional and histological terms, but also elucidate the molecular mechanisms at play, which is indispensable for optimizing material design ahead of clinical translation. Sun et al. [3] developed a composite hydrogel, termed CRP, composed of thermo-sensitive chitosan, self-assembling RADA16 nanofibers, and a neurotropic peptide (PPFLMLLKGSTR), with the aim of recreating a permissive microenvironment for the repair of a completely transected spinal cord in rats. The in situ injection of CRP at the lesion site markedly attenuated the post-traumatic inflammatory response, evidenced by the downregulation of pro-inflammatory cytokines (TNF-α, IL-6) and the upregulation of IL-10, and concurrently inhibited astrocytic hyperproliferation (reduced GFAP) while promoting the migration, proliferation, and neuronal differentiation of endogenous neural stem cells (increased Nestin, TUJ1, and MAP2). These effects translated into significant motor recovery, as assessed by the BBB locomotor scale, with improvements evident from week 2 and sustained through week 8 post-implantation. Molecular analyses implicated the activation of the PI3K/AKT/mTOR pathway as a key mechanism underpinning tissue restoration. Similarly, Pei et al. [65] fabricated an injectable composite consisting of electrospun nanofibers embedded within a self-adjusting hydrogel and loaded with bone marrow-derived mesenchymal stem cells (BMSCs). In a rat model of ischemic stroke induced by middle cerebral artery occlusion (MCAO), the stereotactic delivery of this scaffold led to dramatic reductions in infarct volume and cerebral edema, along with significant attenuation of microglial (Iba-1) and astrocytic (GFAP) activation. Concomitantly, there was enhanced neuronal proliferation (NeuN) and perilesional angiogenesis (CD31), with the formation of new microvessels in the ischemic penumbra. Functionally, treated animals exhibited significantly improved neurological scores by day 7, which persisted through day 28 post-treatment. The therapeutic effect was attributed to paracrine signaling via BMSC-derived exosomes, modulating miR-206-3p expression and activating PI3K/AKT signaling, as confirmed by bioinformatic analysis. An innovative multimodal approach was reported by Yang et al. [64], who combined an aligned fibrin hydrogel (AFG) enriched with magnetic nanoparticles (MAFG) and external magnetic stimulation applied parallel to the spinal axis in rats with complete spinal cord transection. This system promoted macrophage polarization toward the anti-inflammatory M2 phenotype (increased CD206, decreased CD86 and TNF-α), guided axonal alignment and ingrowth, and enhanced both endogenous neurogenesis and angiogenesis. Functionally, MAFG-treated animals under magnetic stimulation demonstrated robust motor recovery, with significantly higher BBB scores from week 2 onward, corroborated by CatWalk gait analysis and motor-evoked potential measurements showing increased amplitude and decreased latency. In a seemingly distinct yet highly relevant domain for neural tissue engineering, Wang et al. [70] designed core–shell coaxial nanofibrous scaffolds with a mesoporous bioactive glass core doped with magnesium and a silk fibroin shell loaded with NGF. In a murine critical-sized calvarial defect model, the implantation of these scaffolds accelerated osteogenesis through the upregulation of Runx2 and the phosphorylation of Erk1/2 and mTOR, while simultaneously promoting neurogenesis, evidenced by newly formed neurons within Haversian canal-like structures in the regenerated bone. Transcriptomic profiling confirmed a synergistic interplay between osteogenic and neurogenic signaling pathways driving “osseoneural” regeneration, accompanied by superior biomechanical restoration and extensive vascular support. The convergence of these experimental paradigms underscores several design principles for next-generation biomaterials: acute inflammatory modulation, the induction of anti-inflammatory cellular phenotypes, the controlled release of neurotrophic factors or differentiation-directing cues, and the optimization of scaffold mechanics and topography to facilitate axonal alignment and neovascularization. Integrating external physical stimuli (electrical, magnetic) or orchestrated paracrine signals from stem cells creates a truly multimodal approach wherein each component synergistically contributes to rebuilding a permissive microenvironment for neural regeneration. Looking toward clinical translation, the next steps should encompass pharmacokinetic and biodistribution studies in larger animal models, long-term safety evaluations, and the refinement of minimally invasive delivery protocols. Only through the integrated analysis of molecular data, functional outcomes, and biocompatibility criteria can the most promising candidates be advanced into Phase I/II human trials. While challenges remain formidable, the robust preclinical in vivo evidence presented here lays a solid foundation for the future development of regenerative therapies targeting conditions that currently lack effective treatment options.

A systematic search of ClinicalTrials.gov identified four early-phase clinical studies investigating the safety and feasibility of electrospun scaffolds combined with stem cells in patients with central nervous system injuries. NCT02688049 is a Phase I/II, randomized, open-label, and parallel-arm trial that enrolled 30 participants with chronic thoracic spinal cord injury (AIS grades A–C). Subjects underwent the surgical implantation of the NeuroRegen Scaffold™ followed by the local administration of 10 × 10^6^ mesenchymal or neural stem cells. Primary endpoints included safety and systemic inflammatory markers at 6 months, while secondary endpoints encompassed ASIA Impairment Scale improvements and motor-evoked potential changes up to 24 months. Enrolment was completed in 2018, with primary completion in December 2021. Similarly, NCT02352077 is a Phase I, open-label, and single-arm study in which 30 patients with chronic spinal cord injury received the same scaffold loaded with 10 × 10^6^ mesenchymal stem cells. Over a 12-month follow-up, no treatment-related adverse events were reported, and preliminary neurophysiological data were collected to inform subsequent efficacy assessments. Extending this approach to intracerebral hemorrhage, NCT02767817 is a Phase I, randomized, double-blind, and parallel-group trial in which 30 patients underwent hematoma evacuation followed by the implantation of a collagen hydrogel scaffold seeded with 10 × 10^6^ mesenchymal stem cells. Across a 24-month observation period, no serious complications or immune reactions were noted, confirming a favorable safety profile. Finally, NCT06361433 (RAINBOW-Hx) is an ongoing Phase I/II, single-group, and open-label study initiated on 1 December 2023. It plans to enrol eight patients with chronic intracerebral hemorrhage sequelae for the stereotactic injection of HUFF-01 (an autologous MSC-loaded scaffold). Safety at 12 months is the primary endpoint, with functional outcomes (mRS) and IMZ-SPECT imaging of the motor cortex as secondary measures; primary completion is expected in February 2026. Together, these four trials mark the first human applications of electrospun scaffold–stem cell constructs, building directly upon the robust preclinical in vivo evidence that supports their translational potential.

## 4. Conclusions and Future Steps

The convergence of electrospun scaffold technologies with mesenchymal stem cell (MSCs) therapy represents a promising and increasingly sophisticated strategy for central nervous system (CNS) regeneration. The studies reviewed consistently demonstrate a synergistic effect between the biomimetic substrate provided by the nanofibrous scaffold and the biological activity of MSCs. While the scaffold physically bridges tissue gaps and guides axonal growth through ECM-like architecture, MSCs contribute through the secretion of neurotrophic and immunomodulatory factors and, to a lesser extent, through differentiation into neural-like cells. This combined approach has proven beneficial in preclinical models of spinal cord injury (SCI), traumatic brain injury (TBI), and stroke, particularly when both structural and biological aspects of the injury microenvironment are simultaneously addressed. Despite this encouraging progress, several translational challenges remain. Another critical issue that emerged from our analysis is the inconsistent reporting of mechanical properties. Only 43% of the reviewed experimental studies provided quantitative parameters such as Young’s modulus, tensile strength, or strain at break. Considering that central nervous system tissues exhibit very soft mechanical characteristics (in the range of 0.1 to 1 kPa), this lack of standardized data prevents meaningful comparison across studies and limits reproducibility. Moreover, scaffold stiffness is a key determinant of MSC fate: softer substrates favour neuronal differentiation, whereas more rigid matrices often direct cells toward glial or osteogenic lineages. For this reason, future research should prioritize the systematic mechanical characterization of electrospun scaffolds through standardized protocols (e.g., uniaxial tensile testing, dynamic mechanical analysis) and the consistent reporting of results. Such standardization would not only improve scientific rigor but also facilitate clinical translation, ensuring that scaffold properties are appropriately matched to the delicate microenvironment of the CNS. Ensuring long-term biocompatibility and seamless integration with host tissue is critical, especially since synthetic polymers may trigger chronic inflammation or fibrosis. Scaffold composition and degradation kinetics must be carefully tuned to avoid mechanical mismatch or toxic byproducts. MSC survival and phenotypic stability also remain hurdles, as many transplanted cells die or migrate away post-implantation, often due to poor vascularization or limited nutrient diffusion. Moreover, donor variability in MSC populations introduces inconsistencies in therapeutic efficacy, necessitating the standardization of cell sourcing and preparation. From a manufacturing standpoint, scaling up electrospun scaffold production while maintaining nanofiber uniformity, sterility, and clinical-grade quality is non-trivial. Additionally, the delivery of the scaffold–cell constructs poses logistical challenges in human CNS injuries, where lesion geometry and surgical accessibility are far more complex than in rodent models. In addition to these biological and manufacturing aspects, several clinical and regulatory challenges remain largely underexplored. One of the major barriers is the immune response elicited by synthetic polymers, which may trigger chronic inflammation or fibrotic encapsulation, complicating long-term scaffold integration. The scalability of electrospinning technologies also poses a hurdle: parameters optimized at the laboratory scale often fail to reproduce identical fiber morphology and properties when transitioned to industrial production. Sterilization is another critical step, as conventional methods such as autoclaving or gamma irradiation can compromise scaffold architecture, mechanical properties, or biofunctionalization. Finally, regulatory approval pathways for advanced combination products that integrate scaffolds, cells, and bioactive factors are complex, requiring the stringent demonstration of safety, reproducibility, and manufacturing consistency. Addressing these translational bottlenecks will be essential to move electrospun scaffold–MSC constructs from preclinical promise toward real clinical application. To address these issues, future directions are moving toward the development of “smart” biomaterials, scaffolds that are not merely passive supports but actively respond to environmental stimuli. These include electroconductive nanofibers capable of delivering bioelectric cues or scaffolds designed to release therapeutic agents in response to inflammation. Gene editing and pre-conditioning strategies to enhance MSC resilience and neurotrophic output are also being explored. Furthermore, a shift toward acellular approaches, leveraging the MSC secretome or exosomes integrated into nanofibers, offers the potential for safer, off-the-shelf alternatives that still capitalize on MSCs’ trophic effects. In particular, the scaffold-mediated delivery of MSC-derived exosomes represents a promising acellular strategy. Optimizing exosome loading methods and fine-tuning release kinetics will be critical to ensure therapeutic efficacy comparable to live MSCs. If these challenges can be addressed, exosome-integrated scaffolds could provide reproducible, safe, and easily deployable treatments for CNS injuries. Notably, MSC-derived exosomes embedded in scaffolds have been shown to promote host cell recruitment and neuroregeneration in vivo, providing a compelling complement or substitute to live-cell therapy. Progress toward clinical translation will also require more advanced preclinical models that better replicate human CNS injury in scale and complexity. Large animal models and humanized organoid systems are being investigated to assess long-term safety and functional integration. Ultimately, the path forward will depend on a multidisciplinary effort, merging materials science, cellular engineering, neurosurgery, and rehabilitation, to not only refine the therapeutic construct but also optimize its delivery and post-implantation support. In summary, electrospun MSC-seeded scaffolds hold considerable promise for driving meaningful neural repair. The foundation has been laid through compelling preclinical evidence showing that scaffolds can support MSC survival and neural differentiation, while guiding axonal regeneration across injury sites. The challenge now lies in refining and integrating this approach into a clinically viable therapy. If successful, such a strategy could represent a paradigm shift in treating CNS trauma and neurodegenerative conditions, transforming the potential of regenerative medicine into functional recovery.

## Figures and Tables

**Table 1:** Comparative summary of 35 selected experimental studies investigating the effects of electrospun biomaterial nanofiber scaffolds on the neural differentiation of mesenchymal stem cells (MSCs) from various tissue sources. Arrows indicate trend direction: ↑ increase, ↓ decrease in the reported level.

Biomaterial (Type/Composition)	Fiber Diameter/Orientation	MSC Source	Growth Factors/Stimulation	In Vitro/In Vivo	MSC Adhesion & Proliferation	Neural-like Differentiation	Mechanical Testing	Reference
IKVAV-PA (self-assembling peptide amphiphile with IKVAV motif)	10.4 ± 1.7 nm; random orientation	Human BM-MSCs (ATCC, P5)	DMEM/F12 + N2/B27 + 20 ng/mL bFGF + 20 ng/mL EGF	In vitro (14 days)	80–90% viability at 24 h; slowed proliferation	Upregulation of Nestin and GFAP at 1 wk; β-III tubulin, MAP2 and NEUN at 2 wk; neuron-like morphology	Storage modulus G′ ≈ 1500 Pa	[54]
CRP composite hydrogel (2% chitosan + RADA16 peptide nanofibers + PPFLMLLKGSTR)	RADA16 nanofibers 1–4 µm; random orientation	Rat BM-MSCs	None (scaffold presents neuro-affinity peptide)	In vitro and in vivo (rat spinal cord injury)	>95% viability; stable proliferation; 97.5% scratch closure at 24 h	Not assessed on MSCs; focus on NSC differentiation and functional recovery in vivo	None reported	[3]
IKVAV-PA hydrogels (C16H31O–A4G3D2–IKVAV)	2–5 nm diameter; 100 nm–1.8 µm length; random network	Rat BM-MSCs (Sprague–Dawley, P3)	None (scaffold provides IKVAV cue)	In vitro only	>95% viability (days 1, 3, 5); proliferation comparable to control	~50% NSE+ neuron-like and ~30% GFAP+ glial-like at 7 days; neuron-like morphology	None reported	[72]
Hybrid PCL/gelatin scaffold (PCL + gelatin + 2% PAG + 2% T3-NPs)	Gelatin/PAG: 263 nm; PCL/T3-NPs: 750 ± 72 nm; random	Rat BM-MSCs → NSCs	Scaffold releases T3 (~25 ng/mL); medium with bFGF, PDGF-AA, heregulin	In vitro only	>95% viability (days 1–3); increased attachment on 2% NP scaffold (days 5–7)	Efficient oligodendrocyte-like differentiation: PDGFR-α+, Olig2+, MBP+; high O4/O1/MOG expression	None reported	[46]
Hydrogel/nanofiber composite (GC/DF-PEG hydrogel + GelMA/PCL core–shell fibers)	Core–shell: 10–300 nm Ø × 100 µm length; random	Rat BM-MSCs	None (oxygen-glucose deprivation stress model)	In vitro and in vivo (rat MCAO model)	>95% viability; enhanced migration; sphere-like aggregation	Paracrine neuroprotection and angiogenesis: ↑ SH-SY5Y viability, neurite outgrowth; ↑ HUVEC tube formation	None reported	[65]
Plasma-treated carbon nanofibers (CNF/Plasma O_2_)	157.5 ± 28.6 nm; random orientation	Human adipose-derived MSCs	Electrical stimulation: 1.5 mA, 500 Hz, 10 min/day; DMEM/F12 + 1% FBS	In vitro only	>90% viability; <7% toxicity; slight proliferative effect	Increased GFAP, MAP2, Nestin and TUJ1 expression under electrical stimulation	None reported	[52]
TPU/MWNT composite scaffolds (TPU + 1.5–3.5 wt% MWNT)	220–350 nm (↓ to 220 nm at 2.5%); random orientation	Rat adipose-derived MSCs	DMEM/F12 + 10% FBS + bFGF + EGF + retinoic acid + BHA; ± EM stimulation	In vitro only	Enhanced adhesion and flattening; increased proliferation with CNT content and EM stimulation	Upregulation of β-III tubulin and MAP2; downregulation of Nestin; accelerated neural differentiation	Tensile strength 5.7 → 20.6 MPa; Young’s modulus 1.8 → 9.8 MPa; decreased elongation	[43]
Collagen-coated PCL nanofibers (electrospun PCL with immobilized collagen)	400–500 nm; random orientation	WJ-MSCs	Pre-induction (24 h): DMEM/F12, 20% FBS, 2% B27, FGF2, IBMX, 2-ME; induction (7 d): DMEM/F12, B27, Shh, RA; maturation (7 d): DMEM/F12, B27, GDNF, BDNF	In vitro (15 d)	Increased adhesion, spreading, and proliferation (days 1–5) vs. controls	Increased motor neuron markers (Islet-1, Pax6, HB9, ChAT); decreased Nestin; and motor neuron maturation	None reported	[16]
Electrospun PCL scaffold (single-polymer PCL)	200–300 nm; random orientation	Human bone marrow and endometrial MSCs	Pre-induction (24 h): DMEM/F12, FBS, B27, FGF2, IBMX, 2-ME; induction (7 d): DMEM/F12, B27, Shh, RA; maturation (7 d): DMEM/F12, B27, GDNF, BDNF	In vitro only	Enhanced adhesion, viability, and proliferation (days 3–5) vs. TCP	Increased motor neuron markers (Islet-1, NF-H, Pax6, HB9, β-III tubulin, ChAT) on PCL vs. TCP	None reported	[7]
Electrospun PCL nanofibers (8% PCL; untreated vs. O_2_-plasma treated)	400–1500 nm; random or aligned; contact angle 130–134° vs. <80°	hMSCs	DMEM/F12 + BDNF + bFGF + NT-3 + NGF + IBMX (15 d)	In vitro (15 d)	Increased adhesion, spreading, and proliferation on p-PCL vs. TCP; random > aligned	Decreased Nestin; increased MAP2 expression; β-tubulin III positive; cells align along fibers	Tensile stress aligned: 24.11→20.97 MPa; random: 1.85→1.68 MPa; strain aligned: 51.4→47.2%; random: 363.8→247.4%	[17]
PCL/gelatin/PRP nanofibrous scaffold (70:30 PCL/gelatin + PRP)	189 ± 56 nm; random	Human scalp adipose-MSCs (P3)	DMEM/F12 + 10% FBS + insulin + indomethacin + IBMX	In vitro only	Gelatin ↑ prolif. vs. PCL; PRP coating further enhances this effect	Nestin and NEUN (early) and MAP2 & TAU (mature) expression; GFAP absent; no difference between scaffolds	None reported	[19]
Wet-electrospun PLA scaffold (15% *w/v* PLA + gelatin/alginate/MWCNT)	Random non-woven with internal pores	WJ-MSCs (P4)	1 mM valproic acid in DMEM/F12 + 10% FBS	In vitro (21 d)	Live/dead: >95% viability; MTT: 91% vs. 76% a 24 h	Nestin, MAP2, and NSE positive; ↑ NeuroD1 & Nestin; ↓ Sox2	None reported	[41]
PCL-gelatin NF microspheres (electrosprayed NF segments)	150–450 µm (varia con voltaggio); random	Rat BM-MSCs	None reported	In vitro only	Adhesion and proliferation increased on nanofibers compared to solid microspheres (*p* < 0.05)	β-III tubulin^+^ extended neurites on NF microspheres; few cells on solid microspheres	None reported	[21]
Magnetic-responsive aligned fibrin hydrogel (5 wt% fibrinogen + PEO + 10 mg/mL Fe_3_O_4_ MNPs)	Aligned (rotor at 50 rpm); diameter not reported	Rat embryonic (E18) NSCs	Magnetic field 200 mT, 3 h/day + EGF (20 ng/mL) & bFGF (10 ng/mL)	In vitro and in vivo	Viability maintained; adhesion and proliferation aligned	↑ Tuj1, NSE, MBP, MOG in vitro; in vivo: ↑ Tuj1^+^ aligned axons, ↑ Nestin^+^/Tuj1^+^ NSC, ↑ synaptophysin and NF200; axonal continuity; improved BBB, CatWalk, and MEP recovery	None reported	[64]
3D oriented carbon nanofiber scaffold (two-nozzle PAN)	Pre-carboniz. 217–343 nm; post 259–797 nm; best at 300 rpm	Mouse BM-MSCs and PC12	None reported	In vitro only	MTT: ↑ viability 24→48 h; LDH/Caspase-3: non-toxic; adhesion superior vs. 2D	Not evaluated (no differentiation marker reported)	None reported	[73]
PLA electrospun scaffold (plasma-treated)	565 ± 18 nm; random	TM-MSCs (trabecular meshwork MSCs)	Neurosphere induction (bFGF, EGF, B27, NEAA) + Schwann induction (forskolin, PDGF-AA, heregulin-β, bFGF)	In vitro (14 d)	High viability; ↑ adhesion vs. TCP; proliferation maintained	↑ S100B, GAP43, GFAP, SOX10; MBP ↑ with serum, ↓ in KOSR; Schwann-like bipolar/tripolar morphology	None reported	[74]
Electrospun PCL/chitosan + in situ Au-NPs (THPC + formaldehyde)	70.8 ± 12.7 nm; random	MSCs (not specified)	Pre-induction (24 h): DMEM/F12 + 20% FBS + 2% B27 + 10 ng/mL FGF2 + 250 µM IBMX + 100 µM 2-ME; induction (9 d): DMEM/F12 + 0.2% B27 + 100 ng/mL SHH + 0.01 ng/mL RA	In vitro only	None reported	57% β-III tubulin^+^ vs. 26% su TCP; ↑ β-III tubulin protein (*p* < 0.05)	Tensile strength 14.47 ± 2.32 MPa; Young’s modulus 127.67 ± 34.51 kPa; and ultimate strain 44.77 ± 6.24%	[22]
Electrospun PCL–PDA nanofibers (dual exosome delivery)	Random porous; diameter similar to PCL NF	hUC-MSC-Exo and mouse NSC-Exo	Local release of dual exosomes (100 µg/mL each)	In vitro (BV2 and PC12) and in vivo	None reported	↓ M1 (CD86, TNF-α, iNOS) and ↑ M2 (CD206, TGF-β, IL-4) in BV2; ↑ migrazione and neuritogenesi PC12; in vivo: ↓ mNSS and miss-step, ↑ rotarod; ↑ GAP-43 and DCX, ↓ GFAP and Iba-1	None reported	[57]
Magnetoelectric PVDF/GO/CFO nanofibrous scaffold	266.9 ± 189.7 nm; random	Human adipose-MSCs (P3)	EM field 1 mT, 50 Hz, 8 h/day; no exogenous factors.	In vitro only	Viability maintained; ↑ proliferation over 21 d (MTT)	↑ Nestin, β-III tubulin & NSE genes; NGFR p75 IF; cell alignment under EM	Tensile strength 6.01 ± 1.02 MPa; strain 12.35 ± 2.89%; Young’s modulus 71.31 ± 6.34 MPa	[71]
PLLA/PCL hybrid nanofibrous scaffold (1:1)	~1.1 µm; allineate (collector 3000 rpm)	TM-MSCs (trabecular meshwork MSCs)	miR-7 overexpression via lentivirus (MOI 15)	In vitro only	Uniform adhesion; viability maintained for 21 d	↑ MAP-2, Nestin and GFAP mRNA a 21 d vs. control; IF conferma ↑ MAP-2 and Nestin	None reported	[23]
Core–shell nanofibrous scaffold: core = Mg-doped mesoporous bioactive glass + OOB; shell = silk fibroin + NGF	Random; diameter not reported	Primary bone-derived MSCs (BMSCs)	NGF (7.2 µg in shell); neurobasal + B27, EGF 20 ng/mL, FGF 20 ng/mL for 7 d	In vitro and in vivo (mouse cranial defect)	Viability ↑ day 1–7	Neurite-like morphology; ↑ β-III tubulin, MAP2, NSE in vitro; new neurons in Haversian canals in vivo	None reported	[70]
0.05% GelMA-coated PCL/0.5% Pluronic F-127 scaffold: PCL (80 kDa) + Pluronic core; 0.05% GelMA coating	Aligned along expansion axis	Rat BMSCs	DMEM + 10% FBS (no added GFs)	In vitro only	↑ adhesion and proliferation day 1–14; viability maintained after minimally invasive delivery	n.a. for MSCs (hNSCs tested separately)	Cyclic compression (50%, 70%, 90% strain; Instron 5640, 9 mm/min): full recovery after 100× 90% cycles; modulus from 0–30% strain; pore-size recovery measured	[24]
PCL nanofibers + DOPA-melanin coating for REST siRNA	Random: 545 ± 9 nm (PCL-RF), 553 ± 13 nm (DM-RF); aligned: 567 ± 12 nm (PCL-AF), 574 ± 13 nm (DM-AF)	Human fetal BM-MSCs	REST siRNA (2–4 µg); neural medium DMEM + 1% FBS + 1% N2 + 1% B27	In vitro only	None reported	↑ Tuj1 (d 7–21); MAP2 by d 14 only on aligned; ↓ GFAP; minimal glial (O4/Olig2); synapsin not detected	None reported	[37]
PCL/GEL bi-electrospun nanofibers: PCL (80 kDa)/Type A Gelatin	PCL: 836 ± 50 nm; PCL/GEL: 407 ± 30 nm; orientation not reported	Human iPSCs (SNL feeder)	Neural induction: DMEM/F12 + 0.5 mM IBMX, forskolin; FBS: 10% (d 1–5), 5% (d 6–10), 2% (d 11–14)	In vitro only	↑ viability on PCL/GEL > PCL > TCPS (d 1–5); >95% at d 3	↑ NSE, MAP2, βIII-tubulin, Olig2, GFAP vs. TCPS; ICC confirmation	Tensile (10 mm/min): PCL σ_u_ = 3.4 ± 0.2 MPa, ε_u_ = 70%; PCL/GEL σ_u_ = 3.2 ± 0.2 MPa, ε_u_ = 25%; Young’s modulus ↑ for PCL/GEL	[33]
PCL-SA nanofiber–hydrogel composite: PCL lattice (600–900 nm) + LMW sulfated alginate (0.004 wt% laminin)	600–900 nm; aligned & random	hMSCs (P5)	Laminin (0.004 wt%); NGF 50 ng/mL in induction medium	In vitro only	DNA content ↑ ~2× vs. PCL (d 7 and 14); viability > 95%	↑ S-100 expression (d 7, 21); neurite extensions	Tensile (20 × 10 mm; Instron 5544, 10 mm/min): modulus 20–35 MPa; max load ↑; suture pull-out 18–20 N; LMW SA > HMW SA; aligned > random	[59]
PPy/PLA composite nanofiber film: PPy nanoparticles embedded in PLA	315.2 ± 3.7 nm; random & aligned	hUC-MSC	DC electrical stimulation 100 mV/mm, 30 min/day × 5 d	In vitro only	↑ adhesion and proliferation vs. PLA film over 5 d	Alignment + ES ↑ NF-L and nestin (~3× vs. TCP); neurite-like outgrowth on aligned + ES	Anisotropic: Along fibers: E = 45.2 MPa, σ_max_ = 4.9 MPa, ε_β_ = 30.7%; ⟂ fibers: E = 14.1 MPa, σ_max_ = 1.3 MPa, ε_β_ = 60%	[13]
PVA/SA electrospun nanofibers (30 wt% SA)	Random; 169 ± 34 nm (30 wt%), 289 ± 66 nm (20 wt%), 488 ± 176 nm (10 wt%) vs. PVA 584 ± 179 nm	hBM-MSCs	β-Carotene 5 and 20 µM in DMEM + 10% FBS for 4 d	In vitro only	Viability ↑ 24 h–21 d (*p* ≤ 0.005)	MAP2 expression and ICC positive at d 7 and 14; neuritic morphology	None reported	[27]
Aligned PCL–collagen I nanofibers (Mb/ADSC ± Schwann cells 1:1:0.5)	Aligned; diameter not reported	Human myoblasts + ADSCs ± Schwann cells	Myogenic medium: DMEM/Ham’s F12 + 2% horse serum + 1 ng/mL bFGF + 0.4 µg/mL dexamethasone + L-glutamine + pen/strep	In vitro only	Good viability over 28 d; no differences in WST-8 or live/dead indices between co-cultures	SC co-cultures ↑ myotube fusion index and MYH2/MYOG vs. Mb/ADSC; aligned multinucleated myotubes	None reported	[31]
Magnetic PLGA nanofibers (0%, 5%, 10% SPION): PLGA (75/25) + SPION	<100 nm; aligned via 3000 rpm rotating collector	hAD-MSC	None; SPION provides magnetic stimulus	In vitro only	Viability and proliferation on 5% and 10% vs. control; 10% > 0%	TUJ-1 ↑ 3.8× (10%) and 1.8× (5%); NSE ↑ 6.3× and 1.2× vs. 0%; ICC confirms ↑ with SPION	Tensile strength 4.08→5.85 MPa (0→10%); elongation ↓; modulus ↑ (20 mm/min)	[39]
Polyaniline–Chitosan nanocomposite (4 wt% PAni in CHI; GA-functionalized)	~35 ± 7 nm (TEM); random	Primary AD-MSC	bFGF and EGF, 10 ng/mL each	In vitro only	Improved viability, adhesion and spreading vs. non-functionalized	>85% βIII-tubulin^+^; ~40% GFAP^+^; pronounced neurite-like projections after 14 d	E = 16–26 kPa; UTS = 347–445 kPa (functionalized) vs. E = 18 kPa; UTS = 379 kPa (non-functionalized)	[67]
PCL/chitosan nanofiber/net + alginate hydrogel microlayer (NT-3 loaded)	Random; ~275 nm thick fibers and ~20 nm ultrathin NFN	Human conjunctiva MSCs (CJMSCs)	NT-3 burst 69% @ 3 d; sustained 90% @ 21 d + DMEM + 10% FBS	In vitro (neural assays); in vivo (subcutaneous)	Alginate coat ↑ CJMSC proliferation vs. unmodified; entrapment design best	RT-PCR: Nestin ↑ 6×; MAP-2 ↑ 5.4×; β-III tubulin ↑ 8.8×; ICC: ↑ Nestin and MAP-2; SEM: neuron-like morphology	PCL/chitosan mat (no coat): thickness 50.3 µm; tensile modulus 53.0 ± 25.9 MPa; UTS 33.1 ± 18.3 MPa; strain at break 1.56 ± 1.08%; yield 0.22 ± 0.07 MPa	[75]
PCL + graphene oxide (0.1 wt% GO) composite nanofibers	PCL: 485 ± 162 nm; PCL+GO: 628 ± 238 nm; random	Human subcutaneous and epidural ADSCs	DMEM + 1% pen/strep, 0.5 mM IBMX, 10 ng/mL BDNF, EGF, bFGF + 20% NSC supplement	In vitro only	GO enhances attachment, proliferation and infiltration vs. PCL alone	Nestin and GFAP expressed in all groups; PCL+GO directs spontaneous MAP2 & CNPase differentiation in epidural ADSCs even without induction medium	None reported	[66]
PCL–PPy conductive nanofiber scaffold	PCL: 492 nm; PCL–PPy: 423 nm; random	CJMSCs	Electrical stimulation 115 V/m, 100 Hz, 1 min/day × 3 days; no added GFs	In vitro only	Viability on PCL–PPy > PCL > TCP at days 3/5/7; ES further enhances viability	qPCR (1 min/day): Nestin ~127×; β-tubulin ~30×; MAP-2 ~52× vs. non-stimulated	Tensile: PCL UTS 25 MPa → PCL–PPy UTS 10 MPa; elongation 60% → 35% (STM-50, 5 mm/min)	[36]
FeOOH/PVDF piezotronic hybrid membrane	PVDF ~600 nm; FeOOH nanorods 80–100 nm × 600–800 nm; random 3D network	Rat BMSCs	Ultrasonic 400 W, 8 min twice daily; no exogenous GFs	In vitro only	CCK-8: viability PVDF ≈ 82% TCP; FeOOH/PVDF slightly lower; US ↑ proliferation	qPCR (21 d + US): Nestin ↑ 89×, Tuj1 ↑ 128×, MAP2 ↑ 220×; no GFAP; GAD65 ↑ 30×, ChAT ↑ 5.5×, DβH ↑ 21×; ICC: Nestin, Tuj1, MAP2; Ca^2+^ imaging: GABA transients (~1.8×)	PFM: d_33_ PVDF 26.8 pC/N; FeOOH/PVDF 27.2 pC/N; ultrasonic piezo-voltage ~2 V	[76]
PCL/gelatin (70:30)/SPION 3D scaffold (wet-electrospun at 350 mT)	605 ± 169 nm; random network under 350 mT	Human olfactory ecto-MSCs (OE-MSCs)	None (no exogenous GFs)	In vitro only	MTT: proliferation on 350 mT > 500 mT scaffolds over 7 d (*p* < 0.05)	RT-PCR (14 d): Nestin ↓; MAP2 ↑ (*p* < 0.05); ICC: β-III tubulin and MAP2; Ca^2+^ imaging: GABA transients confirm functional neurons	Tensile: UTS 0.13 ± 0.06 MPa; Young’s 0.50 ± 0.10 MPa; elongation ~29.6%	[61]

## Data Availability

No new data were created or analyzed in this study. Data sharing is not applicable to this article.

## Abbreviations

The following abbreviations are used in this manuscript:

ACS	American Chemical Society
AD	Alzheimer’s Disease
AD-MSC	Adipose–Derived Mesenchymal Stem Cell
ADSCs	Adipose-Derived Stem Cells
AFG	Aligned Fibrin Hydrogel
AIS	American Spinal Injury Association Impairment Scale
AKT	Protein kinase B
AMP	Adenosine Monophosphate
ASIA	American Spinal Injury Association Impairment Scale
BBB	Blood–Brain Barrier
BDNF	Brain–Derived Neurotrophic Factor
bFGF	Basic Fibroblast Growth Factor
BM	Bone Marrow
BM-MSC	Bone Marrow-Derived Mesenchymal Stem Cell
BMP	Bone Morphogenetic Protein
BMP-2	Bone Morphogenetic Protein 2
BMSC	Bone Marrow Stromal Cell
BMSCs	Bone Marrow Stromal Cells
BV2	Mouse microglial cell line BV2
B27	B27 supplement (neuronal culture supplement)
CD31	Cluster of Differentiation 31 (PECAM-1)
CD86	Cluster of Differentiation 86 (M1 macrophage marker)
CD206	Cluster of Differentiation 206 (M2 macrophage marker)
ChAT	Choline Acetyltransferase
CHI	Chitosan
CiMSCs	Conjunctiva-derived Mesenchymal Stem Cells
CNF	Carbon Nanofiber
CNT	Carbon Nanotubes
CFO	Cobalt Ferrite
CNS	Central Nervous System
CNPase	2′,3′-Cyclic Nucleotide 3′-Phosphodiesterase
CSPGs	Chondroitin Sulfate Proteoglycans
CPs	Conductive Polymers
CRP	C-Reactive Protein
DC	Direct Current
DF-PEG	Difunctionalized Polyethylene Glycol
DMEM	Dulbecco’s Modified Eagle Medium
DOPA	3,4-Dihydroxyphenylalanine
DPSC	Dental Pulp Stem Cell
ECM	Extracellular Matrix
EGF	Epidermal Growth Factor
EM	Electromagnetic
ES	Electrical Stimulation
Exo	Exosomes
FBS	Fetal Bovine Serum
F-127	Pluronic F-127 (Poloxamer 407)
GA	Glutaraldehyde
GAP-43	Growth Associated Protein-43
GC	Glycol Chitosan
GelMA	Gelatin Methacryloyl
GDNF	Glial cell line-Derived Neurotrophic Factor
GFAP	Glial Fibrillary Acidic Protein
GO	Graphene Oxide
HB9	Homeobox 9 transcription factor
hUC-MSC	Human Umbilical Cord Mesenchymal Stem Cell
IBMX	3-Isobutyl-1-methylxanthine
IGF-1	Insulin-like Growth Factor 1
iNOS	Inducible Nitric Oxide Synthase
iPSCs	Induced Pluripotent Stem Cells
IL	Interleukin
IL-4	Interleukin-4
IL-6	Interleukin-6
IL-10	Interleukin-10
IMZ	Imidazole
IMZ-SPECT	Iomazenil Single-Photon Emission Computed Tomography
Iba-1	Ionized calcium binding adaptor molecule 1
Isl-1	Insulin gene enhancer binding protein 1
IJMS	International Journal of Molecular Sciences
IJN	International Journal of Nanomedicine
kPa	kilopascal
LDH	Lactate Dehydrogenase
LMW	Low Molecular Weight
MAP2	Microtubule-Associated Protein 2
MAPK	Mitogen-Activated Protein Kinase
MAFG	Magnetic Aligned Fibrin Hydrogel
MBP	Myelin Basic Protein
MEP	Motor Evoked Potential
mRS	Modified Rankin Scale
miRNAs	MicroRNAs
miR-7	MicroRNA-7
mNSS	Modified Neurological Severity Score
mRNAs	Messenger RNAs
MPa	Megapascal
MSC	Mesenchymal Stem Cell
MSCs	Mesenchymal Stem Cells
MWCNT	Multi-Walled Carbon Nanotubes
mTOR	Mechanistic Target Of Rapamycin
NEUN	Neuronal Nuclear Antigen (NeuN)
Nestin	Intermediate filament protein Nestin
NF-L	Neurofilament Light Chain
NF-H	Neurofilament Heavy Chain
NF200	Neurofilament 200
NGF	Nerve Growth Factor
NGFR	Nerve Growth Factor Receptor
NSE	Neuron-Specific Enolase
NP	Nanoparticle
NSC	Neural Stem Cell
NSCs	Neural Stem Cells
OE-MSCs	Olfactory Ecto-Mesenchymal Stem Cells
OOB	Osteo–Other Bioactive component (core–shell scaffold)
PA	Peptide Amphiphile
PANI	Polyaniline
PC12	Rat pheochromocytoma cell line PC12
PCL	Polycaprolactone
PCL–PDA	PCL-Polydopamine composite
PDA	Polydopamine
PDGF-AA	Platelet-Derived Growth Factor AA
Pen/Strep	Penicillin-Streptomycin
PEDOT	Poly(3,4-ethylenedioxythiophene)
PI3K	Phosphoinositide 3-kinase
PLA	Polylactic Acid
PLGA	Poly(lactic-co-glycolic acid)
PLLA	Poly(L-lactic acid)
PPy	Polypyrrole
PRP	Platelet-Rich Plasma
PVA	Polyvinyl Alcohol
PVDF	Poly(vinylidene fluoride)
RA	Retinoic Acid
REST	Repressor Element 1-Silencing Transcription Factor
REST siRNA	Small interfering RNA targeting REST
RGD	Arginine-Glycine-Aspartic Acid peptide motif
RNA	Ribonucleic Acid
RADA16	Self-assembling peptide (Ac-(Arg-Ala-Asp-Ala)_4_-CONH_2_)
SA	Sodium Alginate
S-100	S-100 protein (glial marker)
SCI	Spinal Cord Injury
SD rat	Sprague-Dawley rat
Shh	Sonic Hedgehog
SDF	Stromal Cell-Derived Factor
SDF-1	Stromal Cell-Derived Factor-1
SNL	SIM mouse fibroblast feeder layer (SNL 76/7 cells)
SPECT	Single Photon Emission Computed Tomography
SPION	Superparamagnetic Iron Oxide Nanoparticle
TCP	Tissue Culture Polystyrene
THPC	Tetrakis(hydroxymethyl)phosphonium Chloride
T3	Triiodothyronine
TBI	Traumatic Brain Injury
TGF	Transforming Growth Factor
TGF-β1	Transforming Growth Factor-β1
TNF	Tumor Necrosis Factor
TNF-α	Tumor Necrosis Factor-α
TPU	Thermoplastic Polyurethane
TUJ-1	Class III β-Tubulin (antibody marker)
TM-MSCs	Trabecular Meshwork Mesenchymal Stem Cells
VEGF	Vascular Endothelial Growth Factor
WJ	Wharton’s Jelly
WJ-MSCs	Wharton’s Jelly Mesenchymal Stem Cells
WoS	Web of Science
